# Shaping the course of early-onset Parkinson’s disease: insights from a longitudinal cohort

**DOI:** 10.1007/s10072-023-06826-5

**Published:** 2023-05-04

**Authors:** Roberta Bovenzi, Matteo Conti, Giulia Rebecca Degoli, Rocco Cerroni, Clara Simonetta, Claudio Liguori, Chiara Salimei, Antonio Pisani, Mariangela Pierantozzi, Alessandro Stefani, Nicola Biagio Mercuri, Tommaso Schirinzi

**Affiliations:** 1 Unit of Neurology, Department of Systems Medicine, University of Rome “Tor Vergata”, Via Montpellier, 00133 Rome, Italy; 2 Department of Brain and Behavioural Sciences, University of Pavia, Pavia, Italy; 3 IRCCS Mondino Foundation, Pavia, Italy; 4 UOSD Parkinson Centre, Tor Vergata University Hospital, Rome, Italy; 5 IRCCS Fondazione Santa Lucia, European Centre for Brain Research, Rome, Italy

**Keywords:** Early-onset Parkinson’s disease, Young-onset Parkinson’s disease, Progression, Gender, Hoehn and Yahr, LEDD, Motor fluctuations

## Abstract

**Introduction:**

Early -onset Parkinson’s disease (EOPD) labels those cases with onset earlier than fifty. Although peculiarities emerged either in clinical or pathological features, EOPD is managed alike typical, late-onset PD. A customized approach would be, instead, better appropriate. Accordingly, a deeper characterization of the clinical course, with an estimation of the disease progression rate, the therapy flow, and the main motor and non-motor complications occurrence, is needed.

**Methods:**

A longitudinal cohort of 193 EOPD patients (selected on a single-centre population of 2000 PD cases) was retrospectively analysed, providing descriptive statics on a series of clinical parameters (genetics, phenotype, comorbidities, therapies, motor and non-motor complications, marital and gender issues) and modelling the trajectories from diagnosis to 10 years later of both Hoehn and Yahr (H&Y) stage and levodopa equivalent daily dose (LEDD).

**Results:**

EOPD had a prevalence of 9.7%, including few monogenic cases. It mostly appeared as a motor syndrome, with asymmetric, rigid-akinetic presentation. H&Y linearly progressed with an increment of 0.92 points/10 years; LEDD flow had a non-linear trend, increasing of 526.90 mg/day in 0–5 years, and 166.83 mg/day in 5–10 years. Motor fluctuations started 6.5 ± 3.2 years from onset, affecting up to 80% of the cohort. Neuropsychiatric troubles interested the 50%, sexual complaints the 12%. Gender-specific motor disturbances emerged.

**Conclusion:**

We shaped EOPD course, modelling a “brain-first” PD subtype, slowly progressive, with non-linear dopaminergic requirement. Major burden mostly resulted from motor fluctuations, neuropsychiatric complications, sexual and marital complaints, with a considerable gender-effect.

## Introduction

Parkinson disease (PD) is a disabling, neurodegenerative disorder due to the loss of dopaminergic nigral cells and the brain accumulation of alpha-synuclein containing Lewy bodies. PD commonly occurs in elderly, and, more rarely, in younger subjects (before the fifty), as early-onset PD (EOPD) [1, 2].

EOPD may include either monogenic forms or, mostly, sporadic cases. If EOPD differs from typical late-onset PD (LOPD) is still matter of debate. There are data suggesting distinctive features for these two conditions, regarding both the pathogenesis and the clinical course [3, 4]. Even in the absence of known gene mutations, the contribution of genetics to EOPD is predominant [5]. As well, the environmental risk factors, the pattern of nigrostriatal denervation, and the load of proteinopathy may differ between EOPD and LOPD [6]. LOPD patients usually have more severe progression, with higher incidence of non-motor disturbances [7]. EOPD patients, instead, may have a more benign disease, but greater frequency of dystonia and levodopa-induced dyskinesia, and, overall, poorer quality of life [3, 8]. Moreover, EOPD women experience remarkable challenges due to menstruation, pregnancy and breastfeeding [9]. However, available data are mostly anecdotal, lacking precise measurements in terms of progression rate, frequency of complications, characterizing elements of the long-term course, which are all fundamental to better comprehend EOPD, and refine a customized approach, overtaking the current equality of treatment between EOPD and LOPD patients.

This study aims to shape EOPD course, from onset to later disease phases, employing real-world data from a single longitudinal cohort, quantifying epidemiological and clinical features, progression of motor disturbances, changes in therapeutic regimen and main complications overall. Establishing such trajectories would be crucial either to improve understanding and management of EOPD patients or construct future observations.

## Materials and methods

### Study population

We performed a retrospective longitudinal study following recent standardized guidelines [10]. Medical charts of 2000 PD patients, diagnosed according to the UK Brain Bank Criteria [11] or the 2015 MDS Criteria [12], and afferent to Tor Vergata University Hospital (Rome, Italy) from 1 January 2000 to 30 November 2021, were screened, and all EOPD patients (AAO ≤ 50 years) enrolled in the study [6, 13].

For each EOPD patient, we collected AOO, gender, family history of PD (presence of at least one affected relative up to second degree), motor phenotype at onset (including the subtype by Rajput et al. [14], the presenting signs, the most affected side), presence of constipation and REM sleep behaviour disorder (RBD) at onset (as defined by the “single-question screen” of Postuma et al., 2012); comorbidities at onset, and diagnostic and care work-up (investigations, time interval between onset and diagnosis, disease duration at first visit in our centre). For patients longitudinally observed, we also collected: the follow-up time; the Hoehn and Yahr (H&Y) score at diagnosis, 5 and 10 years later; data on therapy (initiation treatment, introduction of levodopa); the levodopa equivalent daily dose (LEDD) [15] at diagnosis, 5 and 10 years later; occurrence of motor fluctuations and levodopa induced dyskinesia (LID); occurrence of neuropsychiatric symptoms (depression, anxiety, apathy, psychosis, suicide attempts), cognitive impairment, impulse control disorder (ICD), hallucinations; use of advanced therapies; marital, gender and sexual issues. Genetics was recorded when available.

To allow comparative analysis, a control group of *n* = 184 LOPD patients (AOO > 50 years) diagnosed according the UK Brain Bank Criteria [11] or the 2015 MDS Criteria [12] was also collected from a previously published cohort [16]. For each LOPD patient, we recorded age; gender; AAO; disease duration; comorbidities (vascular risk factors); H&Y score; LEDD; presence of dyskinesia (LID) and presence of neuropsychiatric symptoms, cognitive impairment and visual hallucinations.

Medical charts reviewed for the study were all filled in by movement disorders specialists. Motor and non-motor conditions were diagnosed following current criteria. In particular, for neuropsychiatric disturbances, the DSM-V definitions were used.

The study was conducted in agreement with principles of Helsinki declarations. Local ethical committee approved the study.

### Statistical analysis

Descriptive statics (prevalence, mean, standard deviation calculation) was run on both qualitative and quantitative variables. Frequencies among the groups were compared by Chi square test. Kolmogorov–Smirnov test showed that quantitative variables had a normal distribution. The rate of disease progression, in terms of H&Y score variation over 10 years, was estimated by linear regression. The flow of therapy was also outlined, by using the LEDD variation over 10 years into a quadratic regression analysis, as it better fits compared to a simple linear model. The slope of the regression models was then compared using the *t* test. Correlations analysis was performed by Pearson’s test. Statistical significance was set at *p* < 0.05. Analysis was conducted by means IBM-SPSS 26.

## Results

### Cohort features

We identified 193 EOPD patients on a whole PD population of 2000 cases (prevalence of 9.7%). Age at onset (mean ± st.dev), gender distribution, frequency of main comorbidities and motor features at onset have been summarized in Table 1. Positive family history for PD was reported by 95 patients (49.2%). Diagnosis was made 1.52 ± 1.64 years after first complaints. Additional diagnostic investigations included DaT-SPECT for 44 (22.8%) and CSF analysis for 47 (24.4%). Disease duration (mean ± st.dev) at our first visit/assessment was 5.0 ± 5.07 years; the 88.6% (171/193 patients) have been followed-up for 2.75 ± 4.1 years, with a disease duration at last visit of 9.3 ± 5.8 years.

**Table 1:** Demographics, comorbidities and main motor features at onset of the early-onset Parkinson’s disease (EOPD) study population. In brackets, the sample for which the information was available (percentages are referred to that total value). *n* number of patients, *RA* rigid akinetic, *TD* tremor dominant; *M* mixed

Total *n*
193
Sex
Male: *n* = 107 (55.5%)
Female: *n* = 86 (44.5%)
Age at onset
43.93 ± 5.47 years (range: 28–50 years)
Comorbidities (*n* = 92)
Arterial hypertension: *n* = 29 (15.0%)
Thyroid disorders: *n* = 24 (12.4%)
Back problems: *n* = 12 (6.2%)
Gynaecological disorders: (*n* = 8, 4.2%)
Diabetes, type I or II: *n* = 7 (3.6%)
Malignancy: *n* = 7 (3.6%)
Autoimmune disorders: *n* = 4 (2.1%)
Viral hepatitis: *n* = 4 (2.1%)
Epilepsy: *n* = 4 (2.1%)
Essential tremor: *n* = 2 (1.0%)
Motor phenotype (*n* = 173)
RA: *n* = 90 (52%)
TD: *n* = 67 (37.6%)
M: *n* = 21 (12%)
First motor symptom (*n* = 172)
Tremor: *n* = 65 (37.8%)
Bradykinesia: *n* = 68 (39.6%)
Pain: *n* = 24 (14.0%)
Rigidity: *n* = 11 (6.4%)
Gait difficulties: *n* = 4 (2.3%)
Body distribution (*n* = 180)
Asymmetrical onset: *n* = 171 (95.0%)
Symmetrical onset: *n* = 9 (5.0%)
Dystonia at onset (*n* = 193)
Present in *n* = 24 (12.4%)

In the LOPD group, AAO was 70.2 ± 5.0 years (range 54–87), significantly different from the one of EOPD (*p* < 001). In LOPD patients, male to female ratio was 1.6:1 (60.9% males, 39.1% females), with no significant difference compared to the ratio in EOPD (1.2:1). Mean age and disease duration of the LOPD group were 80.2 ± 3.9 years and 10.0 ± 3.1 years respectively. Regarding comorbidities, diabetes (*n* = 26, prevalence 14.5%) and blood hypertension (*n* = 65, 35.5%) were more prevalent in LOPD than EOPD (*p* = 0.0004 and *p* < 0.0001, respectively). Further details in Fig. 3.

### Motor phenotype at onset and motor progression

According to standard subtypes classification of Rajput et al. [14], 178 out 193 EOPD patients were divided as follows: rigid-akinetic phenotype (RA, *n* = 90, 50.6%), tremor dominant phenotype (TD, *n* = 67, 37.6%), mixed phenotype (M, *n* = 21, 11.8%). Presenting motor signs have been reported in Table 1.

**Fig. 1:**
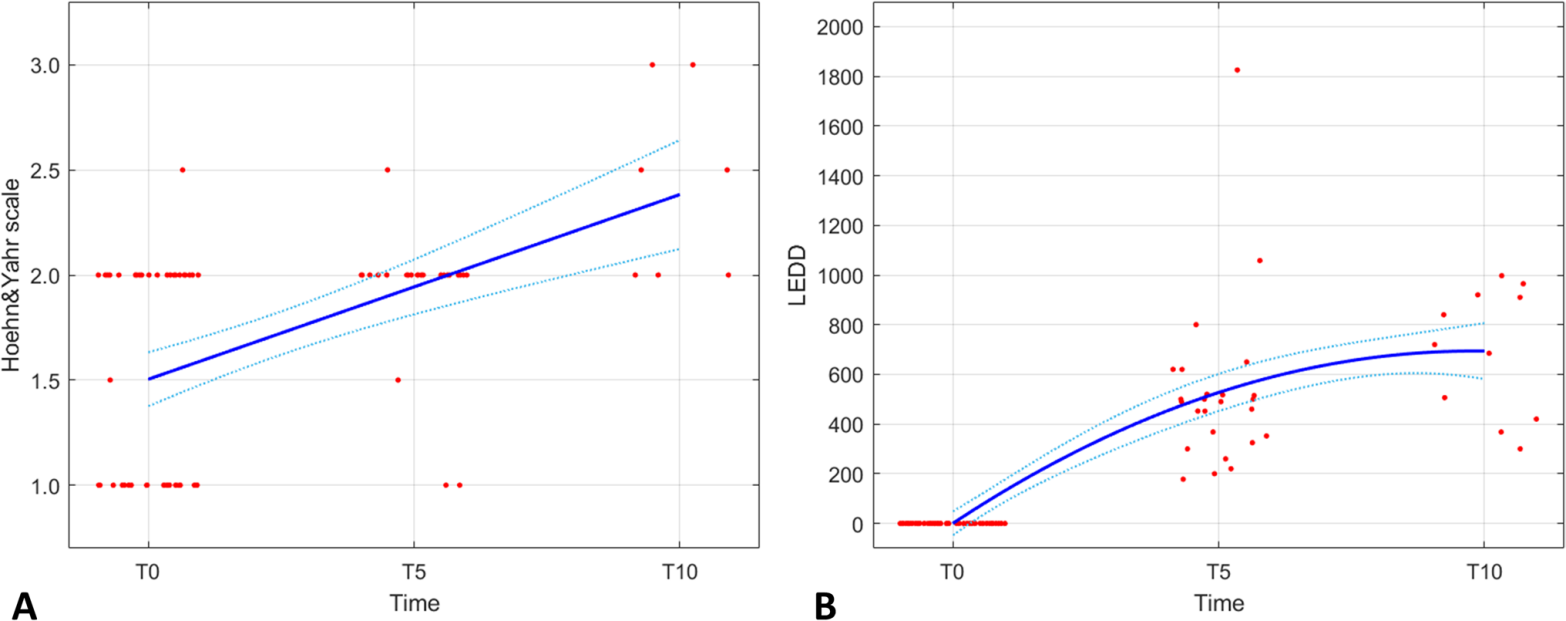
The graphs represent **A** the linear regression of H&Y score (*R*^2^ 0.29, *p* < 0.0001) and **B** the quadratic regression of LEDD (*R*.^2^ 0.70, *p* < 0.0001) over 10 years from diagnosis in the early-onset Parkinson’s disease (EOPD) population. T0 = diagnosis; T5 = five years from diagnosis; T10 = ten years from diagnosis. LEDD, levodopa equivalent daily dose (expressed in mg/day)

For 47 patients, longitudinal H&Y scores at diagnosis (T0), 5 (T5) and 10 years (T10) later were available (H&Y_T0_ = 1.51 ± 0.52; H&Y_T5_ = 1.92 ± 0.32; H&Y_T10_ = 2.43 ± 0.45). Motor progression in terms of H&Y score change over 10 years has been modelled by linear regression, estimating a *R*^2^ coefficient of 0.29 (*p* < 0.0001) (Fig. 1A). *T* test was used to compare slopes between time intervals (H&Y_T5–T0_ = 0.41 [95% CI 0.21 to 0.60], *p* < 0.0001; H&Y_T10–T5_ = 0.51 [95% CI 0.20 to 0.82], *p* = 0.023; H&Y_T10–T0_ = 0.92 [95% CI 0.49 to 1.34], *p* = 0.001).

In LOPD group, H&Y score at 10.0 ± 3.8 years of disease duration was 2.45 ± 0.92. By comparing EOPD H&Y_T10_ score to H&Y score of LOPD patients, no significant differences resulted.

### Non-motor disturbances

At onset, 55 EOPD patients (28.5%) were suffering with RBD and 56 (29.0%) with constipation, mostly in association. Along the entire disease course (from onset to last visit), 50.8% of patients developed neuropsychiatric symptoms, including depression, anxiety, apathy and psychosis. Three patients (1.6%) attempted suicide. Twenty-one patients (10.9%) had cognitive impairment and 16 patients (8.3%) visual hallucinations.

In LOPD group, cognitive impairment occurred in 84 patients (49%) and visual hallucinations in 54 (31.4%), a prevalence significantly higher than in EOPD (*p* < 0.0001 and *p* < 0.0001, respectively). Occurrence of neuropsychiatric manifestations did not differ between the two groups (56.5% in LOPD vs 50.8% in EOPD).

### Therapies and related complications

For 141/193 EOPD patients, data on initiation therapy were available. Patients mostly received dopamine agonists (DA) (*n* = 61, 43.3%, 43 or 70.5% of which with extended-release formulation), monoamine oxidase (MAO) B inhibitors (*n* = 30, 21.3%) or a combination of them (*n* = 31, 21.9%, 22 or 73.3% of which 22 with extended-release DA formulation). Levodopa was the first choice in 19 cases (13.5%), in monotherapy (*n* = 17), associated to DA (*n* = 1) or to MAO B inhibitor (*n* = 1). In general, levodopa was later introduced (4.5 ± 3.8 years from disturbances onset or 2.9 ± 3.3 years from the diagnosis), being finally used in 148/171 patients (86.5% of the longitudinal cohort).

For 60 patients, longitudinal LEDD values at diagnosis (T0), 5 (T5) and 10 years (T10) later were available (LEDD_T0_ = 0; LEDD_T5_ = 526.9 ± 331.5 mg/day; LEDD_T10_ = 693.7 ± 256.3). The flow of therapy in terms of LEDD value changes over 10 years has been modelled by quadratic regression, estimating a *R*^2^ coefficient of 0.701 (*p* < 0.0001) (Fig. 1B). *T* test was used to compare slopes between time intervals (LEDD_T5–T0_ = 526.90 mg/day [95% CI 390.07 to 663.73], *p* < 0.0001; LEDD_T10–T5_ = 166.83 [95% CI − 43.06 to 376.71], *p* = 0.11; LEDD_T10–T0_ = 693.73 [95% CI 521.53 to 865.92], *p* = 0.001). Mean difference between T10 and T5 LEDD was not statistically significant (*p* = 0.11).

In LOPD patients with 10.0 ± 3.8 years of disease duration, LEDD was 833.86 ± 333.34 mg/day, which was significantly higher than LEDD_T10_ of EOPD (LEDD_T10_ LOPD − EOPD = 140.13 mg/day [95% IC 47.41 to 232.84], *p* = 0.003).

Motor fluctuations affected 77.7% of 148 EOPD patients under levodopa, having been appeared 6.5 ± 3.2 years from the disease onset and 1.8 ± 1.7 years from levodopa initiation. LIDs affected 60.8% of 148 levodopa users, 6.7 ± 3.2 years after disease onset and 2.6 ± 2.4 after levodopa initiation. LIDs occurred closer to levodopa initiation in females than males (2.71 ± 1.73 years vs 3.46 ± 2.73 years, *p* = 0.003), but no gender differences resulted when considering the time interval from disease onset (females: 6.50 ± 3.16 years vs males: 7.07 ± 3.42 years, *p* = 0.73).

In LOPD group, 180 patients were treated with levodopa. LIDs occurred in 88 of them (48.9%), a prevalence lower than in EOPD (*p* = 0.03).

**Fig. 2:**
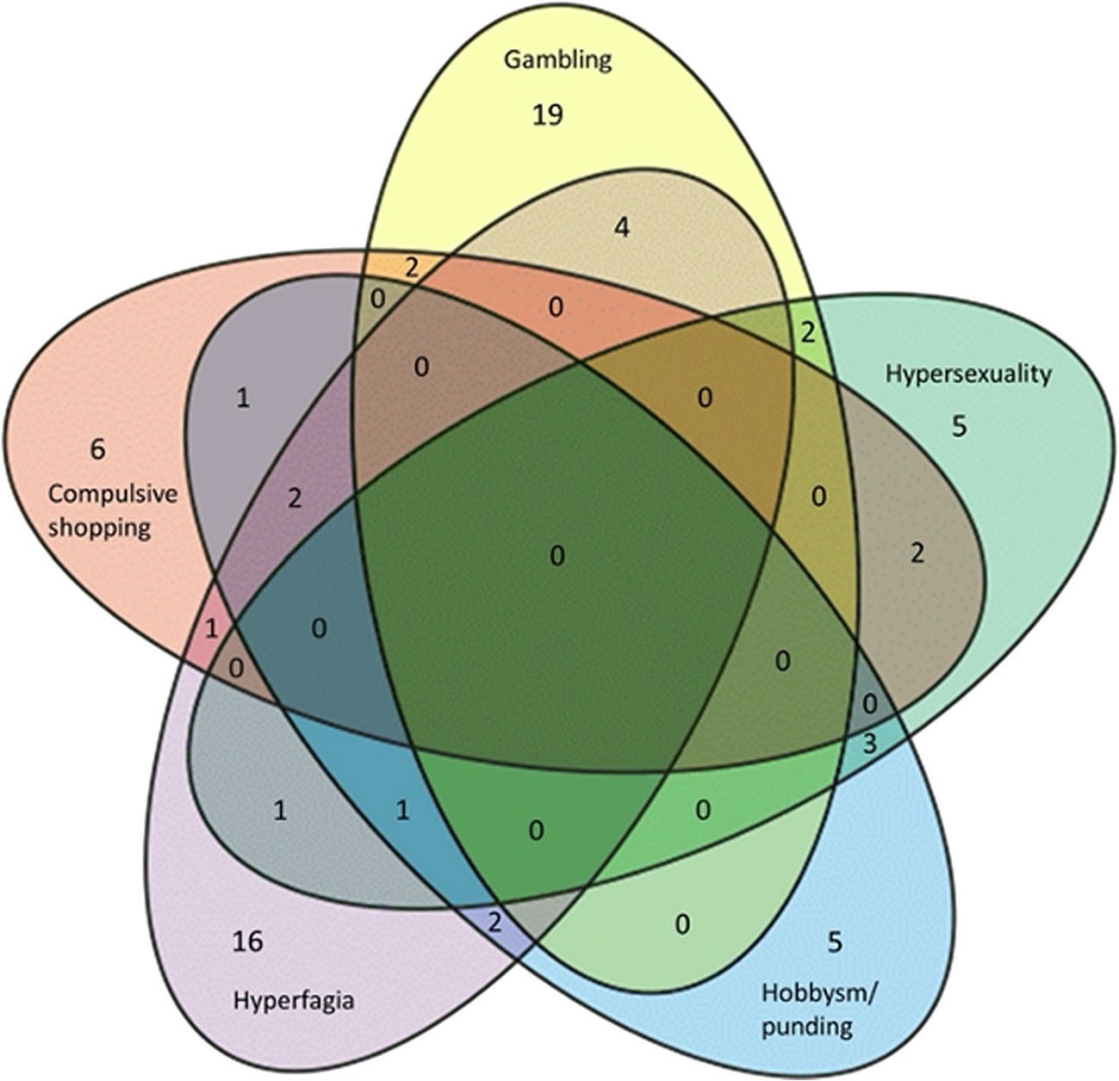
Venn diagram representing the distribution of main ICD manifestations (isolated or in combination) in the early-onset Parkinson’s disease (EOPD) study population. Seventy-two patients (37.3%) of our patients developed ICD, mainly pathological gambling (26.4%) and hyperphagia (22.2%). 29.2% of patients presented a combination of them, especially gambling with hyperphagia (4 patients), hypersexuality and hobbyism (3 patients). ICD, impulse control disorders

**Fig. 3:**
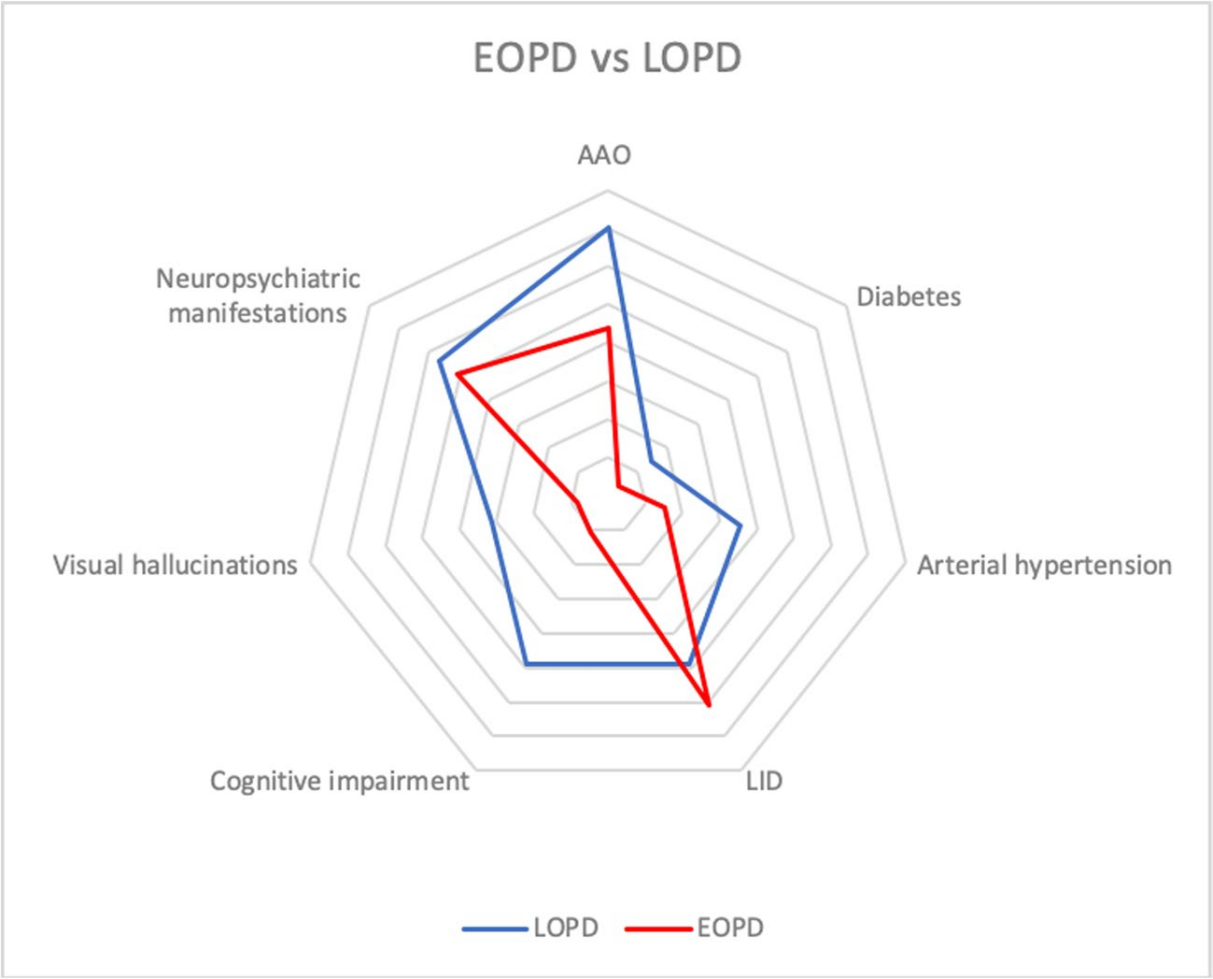
Kiviat diagram representing main clinical differences between EOPD and LOPD patients. EOPD patients have lower cognitive impairment, visual hallucinations and cardiovascular risk factors compared to LOPD. The prevalence of neuropsychiatric manifestations does not differ between the two groups. EOPD patients have higher prevalence of LID than LOPD. EOPD, early onset Parkinson’s disease; LOPD, late onset Parkinson’s disease, AAO, age at onset; LID, levodopa induced dyskinesias

Impulse control disorder (ICD) occurred in 72 EOPD patients, mostly gambling (Figs. 2 and 3).

Advanced therapies (device-aided therapies, DATs) were proposed to 26 out 148 EOPD patients under levodopa (15.2%). Nine refused. The remaining 17 mostly underwent STN-DBS (*n* = 13, 76.5%), while 23.5% received two DATs.

### Marital, sexual and gender issues

Along the disease course, 4/171 EOPD patients divorced (2.3%, male), while two became widowed. Five patients (2.9%) developed a delirium of jealousy (“Othello syndrome”, OS). Sexual difficulties were reported by 24/193 patients (12.4%), namely hypersexuality (*n* = 11, 45.8%), impotence (*n* = 4, 16.7%), reduced sexual desire (*n* = 3, 8.33%) or anorgasmia (*n* = 2, 12.5%). Four patients had both hypersexuality and impotence. One patient attempted unsuccessfully pregnancy. Three out 86 female patients (3.41%) complained with worsening of motor symptoms during menses.

### Genetics

In EOPD patients, a genetic origin was ascertained in 9.3% of cases (18/193 patients). Six patients (33%) were homozygous for pathogenic variants in *PRKN* gene (parkin), six (33%) heterozygous for pathogenic variants in *LRKK2* gene (leucine rich repeat kinase 2), five (28%) heterozygous for pathogenic variants in *GBA* gene (acid beta glucocerebrosidase) and one (6%) homozygous for pathogenic variants in *PINK1* gene (PTEN induced putative kinase 1). Five patients carried variants of unknown significance (VUS).

## Discussion

This study analysed main features of a large, single-centre, longitudinal cohort, to shape EOPD and disclose those elements that might drive a customized approach for such a burdening condition, affecting people in the prime of its life. EOPD is felt as an emerging problem, because of the worldwide increase of frequency [17]. In our population, we estimated a prevalence for EOPD of 9.7% on all PD cases, which, indeed, is greater than expected for Western Counties in early 90 s (5–7% of total PD cases) [18]. Underlying causes of such a slightly higher prevalence cannot be defined here; as well, we can not bear definitely if it reflects a worldwide tendency, rather than some local factor.

The 9.3% of our EOPD cases were pure inherited forms (monogenic PD), in agreement with existing literature [5]. Nevertheless, we found that about 50% of EOPD patients had positive familial PD history, supporting overall the major role of genetics in EOPD pathogenesis. Environmental risk factors have not been explored, but other acquired risk factors, such as diabetes and blood hypertension, had lower rate (3.6% and 15%, respectively) than those observed in our LOPD group, consistent with previous reports [19]. Indeed, EOPD patients are usually considered healthier than LOPD patients [8], albeit only few studies addressed the comorbidity issue [20].

Phenotype at onset of our EOPD patients mostly matched the rigid-akinetic motor subtype (50%), being the tremor-dominant and the mixed one little rarer, as noticed before [7]. PD motor subtypes often underlie differential patterns of circuit disruption [21], thus suggesting a preferential vulnerability of those related to rigidity and akinesia in EOPD. Of relevance, up to 95% of EOPD patients had asymmetric onset, which is now considered a marker of central origin of PD-related neuropathology (“brain-first”), at the opposite of the symmetric onset that, instead, may mark a peripheral, ascending origin (“body-first”) [22]. Moreover, only a minor part of EOPD patients (30%) had concurrent RBD and constipation at onset, two significant hallmarks of “body-first” PD [22, 23], a fact that basically supports the central origin of EOPD. Aside from cardinal motor signs, EOPD patients at onset also presented pain (14%), whereas pure gait difficulties were very uncommon (< 3%).

EOPD patients were diagnosed 1.52 ± 1.64 years after symptoms onset, and mostly received therapy with DAs or MAO B inhibitors (82.5% of patients, in monotherapy or combined). DAs, indeed, represent the most preferred starting treatment and, in general, the most frequently prescribed drugs in EOPD patients [20], although with some regional difference [7]. Levodopa was the first choice treatment only in 13.5% of cases; however, its use increased along the disease course, being progressively introduced 2.9 ± 3.3 years after the diagnosis (or 4.5 ± 3.8 years after onset) in the 86.5% of the whole population. This probably reflects an out-dated tendency to postpone the use of levodopa in order to delay its long-term side effects favouring, instead, the early use of DAs, which, in turn, may induce other kinds of complications and the so-called levodopa phobia [24].

Our EOPD patients have been longitudinally followed-up in average for 2.75 ± 4.1 years, with the first visit 5 ± 5.07 years after onset and the last one 9.3 ± 5.8. Prospective observation allowed us tracking the evolution of motor disturbances, the flow of dopaminergic therapy and the occurrence of a series of non-motor disturbances or disease-related troubles. Precisely, we could estimate on a subgroup of patients the rate of H&Y score progression from diagnosis to 10 years later, modelling a linear growth. We measured that H&Y score increased of 0.41 points in the first 5 years and of 0.51 points in the successive 5 years (0.92 points in the first decade overall). Previous studies, even in naturalistic, real-life contexts, showed that PD patients with typical LOPD basically gain one point of H&Y score every 2 years, with a faster progression in those patients with later or more severe disease onset [25]. EOPD patients, instead, seem to slowly progress in motor impairment, increasing by one H&Y score point over a decade without passing H&Y stage 3, which means preserved postural stability 10 years after onset. Here, we compared the H&Y scores of EOPD and LOPD patients with about 10 years of disease duration, and no significant differences resulted. However, since we did not have baseline H&Y score of LOPD patients and did not follow-up them, we cannot establish if the progression rate was different between the groups.

The flow of dopaminergic therapy, in terms of LEDD value variation, exhibited a non-linear trend (modelled by quadratic regression) in EOPD patients. We observed an early, rapid increase of the dopaminergic dosage (LEDD_T5–T0_ increase = 526.90 mg/day), followed by a substantially slower increase at later phases of the disease (LEDD_T10−T5_ increase = 166.83 mg/day), predicting an overall LEDD of 693.73 mg/day at 10 years from onset. Our LOPD patients had significantly higher LEDD at about 10 years of disease duration, but we could not track the trend over time because of the missing follow-up.

Regarding the therapy flow in EOPD patients, we could suppose that the greater motoric needs due to occupational or familial duties may, at least in part, account for the early, rapid increase. Then, the dopaminergic therapy is more finely tuned at later phases, probably because of the stabilization of motor impairment (as the H&Y score progression may indicate) or, rather, to the occurrence of motor fluctuations, and LID especially. In fact, motor fluctuations overall, and LID specifically, affected up to 80% and 60% of EOPD patients respectively (a percentage significantly higher than LOPD), appearing about 6 years after disease onset or a couple of years from levodopa initiation. The faster LEDD increase might contribute to fluctuations and LID onset of EOPD patients [26], although a sort of vulnerability to early deteriorate the levodopa response has been already described in EOPD compared to LOPD [27, 28], consistent with different patterns of neurodegeneration and compensation [29]. Besides factors related to therapy administration, which have to be considered to prevent or delay disabling motor fluctuations when treating EOPD patients, also other individual or gender-related factors [30] might contribute to the greater occurrence of fluctuations in EOPD, as the development of LID closer to levodopa initiation in female patients suggests. Because of motor fluctuations, advanced device-aided therapies were necessary in 15% of cases, remaining STN-DBS the preferred choice for younger PD patients [31].

Neuropsychiatric symptoms (depression, anxiety, apathy and psychosis) affected up to 50% of patients along the entire disease course, while three patients (1.6%) attempted suicide. EOPD patients are very prone to depression and anxiety [3] and may also have higher suicidal ideation [32]. Accordingly, neuropsychiatric sphere has to be accurately examined and suicidal risk screened in practical management of EOPD patients. Also, ICD frequently affects younger PD patients [33]. In our cohort, ICD, mostly pathological gambling, involved 37.5% of patients, underlining even the need for a constant monitoring of such a dangerous condition, severely impacting on patients and their family. Overt cognitive impairment interested the 10.9% of patients, and visual hallucinations the 8.3%, suggesting that also these major complications are not so uncommon in EOPD, but definitely rarer than in LOPD.

EOPD may also burden on married life and sexuality, although the real prevalence of these problems could be often underestimated, because of embarrassment or discretion in referring to physicians [3]. We found that 2.3% of our patients divorced during the disease course, and 2.9% presented delusional jealousy, namely OS, which increases the risk for divorce in PD. A wide range of sexual difficulties, mostly hypersexuality, has been then reported by 12.4% of our cohort. These, together with other non-sexual factors, such as communication difficulties and mood disturbances, can contribute to affect couple well-being and partner health in EOPD patients [34], accounting for poor quality of life and social or psychological problems in general.

Differently from LOPD, EOPD involves fertile age, complicating menses, pregnancy, birthing and breastfeeding. In our cohort, three female patients complained with worsening of motor disturbances during menses, which is in line with some previous reports [35], although the literature on this topic is almost scarce, highlighting the need for dedicated studies.

This study has several limitations, including the retrospective design and the related bias, some lacking data, the poor assessment of non-motor disturbances and the absence of a control group. We also merged both sporadic and inherited cases, which indeed may exhibit some distinctive trait. Nevertheless, we provided a series of real-world data from a longitudinal cohort supporting a separate approach to EOPD, as a clinical entity distinct from classical LOPD.

## Conclusion

This study provided several useful real-world data regarding EOPD. First, we found that the prevalence of EOPD can be higher than expected from previous epidemiological predictions. Most of the cases were sporadic, although genetic test has not been performed systematically to the entire population. Phenotype at onset of EOPD basically consisted of an asymmetric rigid-akinetic syndrome with a low burden of constipation and RBD, which indeed seems to suggest a central origin of neuropathology (“brain-first” PD subtype). Then, we modelled either the disease progression, showing a slow, linear evolution (0.9 H&Y score points per decade), or the flow of the dopaminergic therapy, outlining a non-linear progression with a faster LEDD increase in the first years of the disease (526.90 mg/day) and a milder increase later (166.83 mg/day), probably reflecting motoric needs and complications. Therefore, we drew a clinical course typically characterized by motor fluctuations, neuropsychiatric disturbances, ICD and other sexual or marital troubles. Finally, we provided some comparisons with LOPD patients, showing peculiar differences regarding the therapy, the complications and the comorbidities.

All these data might be relevant to develop a customized approach to EOPD. In particular, we provided meaningful information for possible trial or preventive strategies design, including the progression rate of motor impairment and therapy flow, as well as the potential “brain-first” origin of EOPD. Then, we identified several critical hints to drive therapeutic choices, such as prevalence of motor and non-motor complications and the need for adequate psychological support for social and familial issues. On the other hand, further themes deserving appropriate investigations emerged, such as those related to pathogenesis or gender differences.

## Data Availability

Data is available from the corresponding author upon reasonable request.
